# Evaluation of the diagnostic accuracy of lateral flow devices as a tool to diagnose rabies in post-mortem animals

**DOI:** 10.1371/journal.pntd.0008844

**Published:** 2020-11-05

**Authors:** Kazunori Kimitsuki, Nobuo Saito, Kentaro Yamada, Chun-Ho Park, Satoshi Inoue, Motoi Suzuki, Mariko Saito-Obata, Yasuhiko Kamiya, Daria L. Manalo, Catalino S. Demetria, Milagros R. Mananggit, Beatriz P. Quiambao, Akira Nishizono

**Affiliations:** 1 Department of Microbiology, Faculty of Medicine, Oita University, Yufu, Oita, Japan; 2 Laboratory of Veterinary Public Health, Department of Veterinary Medical Science, Faculty of Agriculture, University of Miyazaki, Miyazaki, Miyazaki, Japan; 3 Department of Veterinary Pathology, School of Veterinary Medicine, Kitasato University, Towada, Aomori, Japan; 4 National Institute of Infectious Disease, Tokyo, Japan; 5 Tohoku University Graduate School of Medicine, Sendai, Miyagi, Japan; 6 School of Tropical Medicine & Global Health, Nagasaki University, Nagasaki, Nagasaki, Japan; 7 Research Institute for Tropical Medicine, Muntinlupa City, Metro Manila, Philippines; 8 Regional Animal Disease Diagnostic Laboratory, Department of Agriculture Field Office III, San Fernando, Pampanga, Philippines; Centers for Disease Control and Prevention, UNITED STATES

## Abstract

Implementation of lateral flow devices (LFDs) for rabies antigen detection is expected to improve surveillance through the efficient detection of rabid animals in resource-limited settings; however, the use of LFDs for diagnosis remains controversial because some commercially available kits show low sensitivity. Therefore, we compared the diagnostic efficacy of three LFDs (ADTEC, Bionote, and Elabscience kits) paralleled with the direct fluorescent antibody test (dFAT) using fresh samples and investigated the diagnostic accuracies. To do so, we evaluated rabies-suspected samples submitted to the Regional Animal Disease Diagnostic Laboratory III, Philippines. Furthermore, we conducted real-time RT-PCR and sequencing to measure the accuracy of field laboratory diagnosis. The total number of animals submitted during this study period was 184 cases, including negative control samples. Of these, 53.9% (84 cases) were positive in the dFAT. Dogs were the most common rabies-suspected animal (n = 135). The sensitivities of the ADTEC and Bionote kits were 0.88 (74 cases) and 0.95 (80 cases), respectively. The specificity of both kits was 1.00 (100 cases). Furthermore, the sensitivity and specificity of the ADTEC kit after directly homogenizing the samples in assay buffer without dilution in phosphate-buffered saline (ADTEC kit DM) were 0.94 (79 cases) and 1.00 (100 cases), respectively. By contrast, there were no positive results using the Elabscience kit among all dFAT-positive samples. The sensitivity and specificity of LFDs make these tests highly feasible if properly used. Therefore, LFD tests can be used to strengthen the surveillance of rabies-infected animals in endemic and resource-limited settings.

## Author summary

Implementation of lateral flow devices (LFDs) for rabies antigen detection is expected to improve surveillance through the efficient detection of rabid animals in resource-limited settings because they are not only accurate but rapid, user-friendly, and low-cost tools. Although LFDs may positively contribute to accurate data reporting, the use of LFDs for definitive diagnosis remains controversial because some LFDs showed inadequate ability. In this study, we evaluated the diagnostic accuracy of three LFDs (ADTEC, Bionote, and Elabscience kits) paralleled with the direct fluorescent antibody test (dFAT) and real-time RT-PCR using fresh samples and investigated discrepancies among these tests. Of these, half of the submitted samples were positive in the dFAT. The sensitivity and specificity of the simplified ADTEC method (ADTEC kit DM) and Bionote kits were comparable with those of dFAT, but no positive reactions were observed using the Elabscience kit. Despite controversial discussions regarding the use of LFDs, we demonstrated that the diagnostic accuracy of ADTEC and Bionote kits was satisfactory under regional diagnostic laboratory conditions. Our results support the potential of LFD tests for in-field diagnosis of rabies in endemic countries and the feasibility for practical use when they are properly used.

## Introduction

Rabies is caused by lyssaviruses, among which the rabies virus acts as the main etiological agent of human rabies. Once symptoms appear, the disease is incurable and causes inevitable death following encephalomyelitis. Approximately 59,000 individuals die annually because of rabies; 95% of these belong to developing countries in Asia and Africa [1,2]. In the previous decades, there has been a dramatic decrease in the number of dog-mediated human rabies cases in the western hemisphere because of effective mass dog vaccination in addition to the control of the dog population [3–5].

Controlling rabies in dogs is essential to eradicate dog-mediated human rabies deaths [6]. As the World Health Organization (WHO), the Food and Agriculture Organization of the United Nations, the World Organization for Animal Health (OIE), and the Global Alliance for Rabies Control seek to accelerate their actions toward the elimination of dog-mediated rabies by 2030, they are presently joining forces to support countries [7–9]. Mass dog vaccination is included in the first phase of this activity because it is the most cost-effective strategy to control and eventually eliminate rabies. To implement mass vaccination in resource-limited countries, it is necessary to design a less expensive but effective vaccination program [10,11]. Surveillance and epidemiology data are essential to design effective and economic strategies, such as focusing on areas where rabies is endemic. A rapid, user-friendly, and low-cost method to detect rabid animals may increase the collection of reliable data of confirmed rabies cases. Consequently, it might contribute to the improvement of surveillance that is lacking in the majority of rabies-endemic countries.

The direct fluorescent antibody test (dFAT) is one of the standard diagnostic methods for rabies diagnosis, which has been internationally approved by the OIE and the WHO [2,12]. However, the fluorescent antibody test, as a reference test, is not practicable in several endemic settings because of the costs of animal dissection facilities, instruments, equipment such as fluorescence microscopes and incubators, and enhanced laboratory requirements and technical expertise for interpretation of the results [13,14]. In addition to this, acquiring the associated reagents such as fluorescein isothiocyanate (FITC) conjugate may be difficult in low- or middle-income countries. In the previous decade, the OIE and WHO recommended the direct rapid immunohistochemical test (dRIT) and detection of viral genomes by RT-PCR as reliable methods [2,12]. dRIT is faster than dFAT and does not require a fluorescence microscope. It is expected that dRIT can promote decentralized surveillance in developing countries [2,15]. However, a significant concern is that conjugated monoclonal antibodies that are not commercially available are obtained only through a few laboratories specialized in rabies diagnosis [14]. Another major limitation of the dRIT approach is that it requires hazardous chemical reagents for fixation and needs to maintain a cold chain to store the conjugate. RT-PCR can be applied to any sample condition even with decomposed tissue that cannot be used with ordinary viral antigen detection (dFAT and dRIT) [16,17]. However, this method requires high-quality assurance and experience, which are challenging to implement continuously in regional diagnostic laboratories, where human resources and devices are lacking [14]. In recent years, several studies have reported the application of lateral flow devices (LFDs) as a substitute for other rabies diagnostic methods [18–36]. In practice, the use of LFDs has some advantages compared to dFAT, dRIT, and RT-PCR, which are usually performed in central laboratories [14], because several companies provide ready-to-use kits. Therefore, it is expected that LFDs are readily available, even in developing countries [28].

Most of the recent studies have shown that the accuracy of LFDs, especially the Bionote kit, is comparable to dFAT; however, the use of LFDs for diagnosis remains controversial because some of the commercially available kits have demonstrated only a limited positive detection power and insufficient results in multicenter studies [20,29,37]. These studies used stored samples for the assessment of LFDs [29,37]; therefore, their sensitivity might vary considerably depending on the sample conditions. Previous studies evaluating Bionote kits suggested that fresh samples collected in the field provided higher sensitivity values than long-term stored samples [29,37]. In our previous study using the ADTEC kit developed in 2008, which is not included in the evaluation of commercially available kits in the study by Klein et al. [37], we found that the sensitivity varied from 0.74 to 0.95, depending on the animal species when using preserved samples in a multicenter study [18,19].

Therefore, it is necessary to evaluate the use of fresh samples in practical situations to compare LFD accuracies. To our knowledge, there have been no reports evaluating multiple kits using fresh samples collected at an actual diagnostic laboratory. In this study, we assessed the diagnostic performance of three available LFDs (Bionote, ADTEC, and Elabscience kits) paralleled with dFAT using fresh samples collected prospectively at a regional animal diagnostic laboratory in an area of the Philippines, where approximately 200 people die annually because of dog-mediated rabies.

## Methods

### Ethical statement

In the Philippines, RADDL collects personal information as a routine practice for national surveillance. In this study, we used only this information after excluding any individual identifiable information. We obtained verbal informed consent to use the information for our research. Because we only collected samples from carcasses or animal heads submitted from civilians or organizations, the Institutional Animal Care and Use Committee waived the animal ethical approval. For biosafety clearance, our research protocol was approved by the Biosafety Clearance of RITM (No.190116).

### Study site

We conducted a prospective study to investigate the accuracy of the three LFDs at the Regional Animal Disease Diagnostic Laboratory III (RADDL III), a government agency in the Philippines where suspected rabies specimens are submitted from areas throughout Central Luzon (Region III). Region III has a population of 11,218,177 (2015 census) [38]. The number of human rabies infections officially reported in Region III in 2018 was 58 cases, with 252 cases of animal rabies, which was one of the highest numbers in the Philippines [39].

### Data and sample collection

When an individual submitted the head of a rabies-suspected animal, the research staff collected detailed information on the samples and performed dFAT and LFD as soon as possible within a day. Some of the brain specimens of the same animal were stored at −80°C in an ultra-low-temperature freezer and sent to the Research Institute for Tropical Medicine (RITM) in Manila for further analysis.

### Sample collection

We enrolled all rabies-suspected animals that were euthanized or had naturally died and were submitted to RADDL III from all provinces in Region III between 21^st^ April 2019 and 30^th^ November 2019. RADDL III generally conducts surveillance to investigate the number of rabid animals among stray dogs being caught for dog population control. Of these, the cases that were confirmed as rabies negative by dFAT were included as negative controls (non-suspected rabid animals).

### Data collection

The research staff conducted semi-structured interviews to collect detailed information about the animal, owner, and bite victims using a standard questionnaire created for this research. When no sufficient information was available at the time of sample submission, the research staff conducted telephone interviews with the owner to obtain additional information required for the research. The research staff entered the collected data into a system (REDcap Consortium, Nashville, TN, USA) without any personal identifiable information. Another staff member double-checked the data to avoid any errors.

### Sampling method and diagnosis

The laboratory staff collected the hippocampus, cerebellum, and brain stem as soon as possible after submission and used them for definitive diagnosis and LFD. The RADDL III staff performed dFAT as the definitive diagnosis, and the research staff performed the LFDs at the same time. Another RADDL III staff member, who was not involved in the rabies diagnosis, read the LFD results. To prevent any possible interference between the dFAT and LFD results, staff members read the LFD result blindly, and a different member of staff stored the dFAT and LFD results in the data system. The hippocampus and cerebellum specimens were used for dFAT, and the brain stem was used for both dFAT and LFD. These tissues were separately collected and stored for further analysis.

### dFAT

In all cases, dFAT was performed as the reference test. Briefly, touch impressions of small transverse sections (2–3 mm in thickness) of the hippocampus, brain stem, and cerebellum were stained with fluorescein isothiocyanate-conjugated anti-rabies monoclonal antibody (Fujirebio, Malvern, PA, USA; lot No. 309303) according to the standard operating procedure [40]. Then, they were examined under an epifluorescence microscope (E200, Nikon, Tokyo, Japan) to confirm the presence of the rabies virus antigen by two independent examiners.

### Lateral flow devices

The LFDs used in this study were selected based on previous literature and internet searches. Of the commercially available kits, the Anigen Rapid Rabies Ag test kit (Bionote, Inc, Hwaseong, Korea; lot No. 1801DD025), Rabies Ag test (ADTEC Co., Ltd., Oita, Japan; lot No. 1904), and Rabies Virus Antigen Rapid Test Kit (Elabscience Biotechnology, Inc., Wuhan, China; lot No. A6QYBVSSXI) were purchased. While the ADTEC and Bionote kits have already been evaluated in several studies, Elabscience has only been used to compare the sensitivity and specificity of several commercial LFD kits [37]. Approximately 1 g of tissue obtained from the brain stem was homogenized with 500 μL phosphate-buffered saline (PBS) using a BioMasher II (Nippi Inc., Tokyo, Japan). The homogenate was collected using a swab and then mixed with the assay buffer packaged in the kit until the sample was completely dissolved. The supernatants were dropped into the sample hole using a disposable dropper (4 drops, approximately 120 μL), and the results were read after 15 min. The sample processing methods of the three LFDs in this study were quite similar regarding the protocol for preparing brain homogenates by the addition of PBS. However, Léchenne et al. reported that skipping the dilution step of the brain sample in PBS resulted in satisfactory results [33]. Therefore, we also skipped the dilution step of the brain sample by PBS and directly homogenized it using the assay buffer and applied it to the ADTEC kit (ADTEC kit DM). The ADTEC kit DM was conducted at RITM using identical stored samples from RADDL III. Four independent examiners blindly judged the results of the LFDs under the same conditions (room lighting and reaction time) without knowing the results of other tests, including dFAT, and the test result was recorded as a digital image to enable subsequent verification. All examiners underwent the same training to distinguish between positive and negative results before starting this study.

### RNA extraction and real-time RT-PCR

Total RNA was extracted from the frozen brain stem using the High Pure RNA Tissue Kit (Roche Molecular Biochemicals, GmbH, Mannheim, Germany). Approximately 30 mg of tissue was homogenized using a homogenization pestle, and RNA was extracted according to the manufacturer’s recommendations. Then, 50 μL of RNA was eluted and stored at −30°C in a low-temperature freezer until further use. For real-time RT-PCR, the LN34 assay was performed using AgPath-ID One-step RT-PCR Reagents (Applied Biosystems, Foster City, CA, USA) [41–43]. The master mix consisted of the following: 6.5 μL of ddH_2_O, 12.5 μL of 2× RT buffer, 1 μL of 25× RT-PCR Enzyme Mix, 1 μL of either LN34 or beta-actin primer sets (10 μM), 1 μL of either LN34 or beta-actin probe (5 μM), and 2 μL of RNA template [41–43]. The sealed plate was placed into an ABI Step One Plus Real-Time PCR (Applied Biosystems Foster City, CA, USA), and the following conditions were set: reverse transcription at 50°C for 30 min, denaturation at 95°C for 10 min, and amplification of 45 cycles at 94°C for 15 s and 56°C for 30 s using ABI7500-standard mode. To estimate viral load, the Cq values were divided into >25 (low copy numbers), 15–25 (high copy numbers), and <15 (very high copy numbers).

### Nucleotide sequencing

The rabies virus N gene was amplified using the primers p1 and 304 using superscript III One-Step RT-PCR with Platinum Taq Polymerase (Invitrogen, Carlsbad, CA, USA), which generated an amplicon of 1,506 bp [44]. The amplified DNA products were visualized under UV transillumination after electrophoresis using SYBR Safe Gel (Invitrogen)-stained agarose gels. The PCR products of the discrepant samples were subjected to Sanger sequencing, and data analysis was conducted using MEGA X [45]. In addition to the aforementioned primer sets, a cocktail of JW6 DPL (Duvenhage virus PV and Lagos bat virus), JW6 M (Mokola virus), and JW6 E (EBLs 1 and 2) primers was used for sequencing [46]. To construct a phylogenetic tree, neighbor-joining phylogenetic analysis was performed using the Kimura-2 parameter in MEGA X. Bootstrap support was estimated for 1,000 replicates.

### Data analysis

The analysis included the diagnostic accuracy of ADTEC, Bionote, Elabscience kits, and ADTEC kit DM to detect rabies antigen in animal brain samples compared with dFAT or LN34 real-time RT-PCR assay. Sensitivity, specificity, and positive and negative predictive values (PPV and NPV, respectively) of each kit compared with those of dFAT or real-time RT-PCR were determined using 2 × 2 contingency tables. The 95% confidence intervals (CIs) were calculated for the variables being analyzed. The concordance between rabies detection tests was evaluated using the Kappa test and the McNemar test. The kappa value of agreement levels was interpreted as no agreement (<0), slight agreement (0.00 to 0.20), fair agreement (0.21 to 0.40), moderate agreement (0.41 to 0.60), substantial agreement (0.60 to 0.80), and almost perfect agreement (0.80 to 1.00). Only one sample (ID 0075) showed invalid results for LFD and was therefore excluded from all statistical analyses. The final rabies-suspected samples included, thus, 156 cases. The parameters were computed using GraphPad Prism 8 (GraphPad Software, CA, USA). Statistical analyses were performed using Stata software (version15; StataCorp, College Station, Texas).

## Results

### Performance of the ADTEC, Bionote, and Elabscience kits compared with that of dFAT in RADDL III and evaluation of a simplified processing method for the ADTEC kit

During the study period, 156 rabies-suspected animals were subjected to rabies testing (S1 Table and S2 Table). The submitted samples included 135 dogs (67 males, 46 females, and 22 unknowns), 20 cats (5 males, 6 females, and 9 unknowns), and one monkey (male; S2 Table). Among rabies-suspected animals, 84 cases (53.9%) were positive in the dFAT. In addition to these samples, 28 dog heads that were not suspected of having rabies were also examined by LFD analysis (total 184 samples; S1 Table). Compared with dFAT, the sensitivities of ADTEC and Bionote kits were 0.88 (CI = 0.80–0.93) and 0.95 (CI = 0.88–0.98), respectively, and the specificity of both kits was 1.00 (CI = 0.96–1.00). On the other hand, no positive cases were found using the Elabscience kit (Table 1). The ADTEC and Bionote kits identified 10 and 4 false negatives, respectively. In the ADTEC kit DM, there were 5 false-negative results with a sensitivity of 0.94 (CI = 0.87–0.97), and the specificity was 1.00 (CI = 0.96–1.00). The kappa value indicated that the ADTEC kit, Bionote kit, and ADTEC kit DM showed almost perfect agreement (Table 1). The exact McNemar significance probability between the Bionote kit and the ADTEC kit was 0.03, but no significant difference was observed between the Bionote kit and the ADTEC kit DM (exact McNemar significance probability = 1.00).

**Table 1:** Sensitivity, specificity, positive predictive value (PPV), and negative predictive value (NPV) of each kit compared with those of the direct fluorescent antibody test (dFAT).

		dFAT		Sensitivity (95% CIs)	Specificity (95% CIs)	PPV (95% CIs)	NPV (95% CIs)	Kappa value (95% CIs)	
		Positive	Negative						
**ADTEC kit**	**Positive**	74	0	0.88 (0.80–0.93)	1.00 (0.96–1.00)	1.00 (0.95–1.00)	0.91 (0.84–0.95)	0.89 (0.82–0.95)	Almost perfect agreement
	**Negative**	10	100						
**Bionote kit**	**Positive**	80	0	0.95 (0.88–0.98)	1.00 (0.96–1.00)	1.00 (0.95–1.00)	0.96 (0.91–0.99)	0.96 (0.91–1.00)	Almost perfect agreement
	**Negative**	4	100						
**Elabscience kit**	**Positive**	0	0	0.00 (0.00–0.04)	1.00 (0.96–1.00)	NR	0.54 (0.47–0.61)	0.00 (0.00–0.00)	No agreement
	**Negative**	84	100						
**ADTEC kit DM**	**Positive**	79	0	0.94 (0.87–0.97)	1.00 (0.96–1.00)	1.00 (0.95–1.00)	0.95 (0.89–0.98)	0.95 (0.90–0.99)	Almost perfect agreement
	**Negative**	5	100						

dFAT: direct fluorescent antibody test, PPV: positive predictive value, NPV: negative predictive value, NR: not rated, 95% CI: 95% confidence interval

### Further molecular analysis of field samples and analysis of samples with discrepant dFAT and LFD results

According to the data on the sensitivity and specificity of LFDs, false-negative cases were found in LFD tests with dFAT as the reference test (ADTEC kit: 10 cases, Bionote kit: 4 cases, Elabscience kit: 84 cases, and ADTEC kit DM: 5 cases; Table 1). Next, we examined the viral copy number of the tested samples and found that dFAT showed a sensitivity of 0.98 (CI = 0.92–1.00) and specificity of 0.99 (CI = 0.95–1.00), compared to the real-time RT-PCR (PPV = 0.99, NPV = 0.98; Table 2). Similarly, the sensitivities of the LFDs ADTEC kit, Bionote kit, and ADTEC kit DM were 0.87, 0.94, and 0.93, respectively, compared to the real-time RT-PCR results (Table 2).

**Table 2:** Sensitivity, specificity, positive predictive value (PPV), and negative predictive value (NPV) of each test compared with those of the real-time RT-PCR.

		Real-time RT-PCR		Sensitivity (95% CI)	Specificity (95% CI)	PPV (95% CI)	NPV (95% CI)	Kappa value (95% CIs)	
		Positive	Negative						
**dFAT**	**Positive**	83	1	0.98 (0.92–1.00)	0.99 (0.95–1.00)	0.99 (0.94–1.00)	0.98 (0.93–1.00)	0.97 (0.93–1.00)	Almost perfect agreement
	**Negative**	2	98						
**ADTEC kit**	**Positive**	74	0	0.87 (0.78–0.93)	1.00 (0.96–1.00)	1.00 (0.95–1.00)	0.90 (0.83–0.94)	0.88 (0.81–0.95)	Almost perfect agreement
	**Negative**	11	99						
**Bionote kit**	**Positive**	80	0	0.94 (0.87–0.98)	1.00 (0.96–1.00)	1.00 (0.95–1.00)	0.95 (0.89–0.98)	0.95 (0.90–0.99)	Almost perfect agreement
	**Negative**	5	99						
**Elabscience kit**	**Positive**	0	0	0.00 (0.00–0.04)	1.00 (0.96–1.00)	NR	0.54 (0.47–0.61)	0.00 (0.00–0.00)	No agreement
	**Negative**	85	99						
**ADTEC kit DM**	**Positive**	79	0	0.93 (0.85–0.97)	1.00 (0.96–1.00)	1.00 (0.95–1.00)	0.94 (0.88–0.97)	0.93 (0.88–0.97)	Almost perfect agreement
	**Negative**	6	99						

dFAT: direct fluorescent antibody test, PPV: positive predictive value, NPV: negative predictive value, NR: not rated, 95% CI: 95% confidence interval

We further analyzed the characteristics of 12 discrepant samples by comparing the results with those of the real-time RT-PCR as the standard criterion. ID 0132 and ID 0140 showed positive Cq values (31.04 and 31.43, respectively) of viral copy numbers despite being negative in the dFAT, indicating false-negative dFAT results. ID 0156 showed no Cq value despite being positive in the dFAT, suggesting a false-positive dFAT result. Although ID 0155 and ID 0157 showed positive Cq values (33.07 and 33.42, respectively) by real-time RT-PCR, neither ADTEC nor Bionote kit showed positive results, indicating false negatives in these two LFDs (Table 3). Apart from these five cases, the remaining seven cases (IDs 0055, 0066, 0087, 0093, 0121, 0130, and 0131) showed discrepant results between LFDs (either ADTEC or Bionote), and the real-time RT-PCR returned high viral copy numbers according to the Cq value. Except for the Elabscience kit, the kappa value of dFAT or LFD tests and real-time RT-PCR indicated almost perfect agreement (Table 2).

**Table 3:** Results of the 12 discrepant samples.

Study ID	Real-time RT-PCR	Cq value	dFAT	ADTEC kit	Bionote kit	ADTEC kit DM	Elabscience kit	Sequence determined
**0055**	+	20.11	+	-	-	-	-	Yes
**0066**	+	14.81	+	-	+	+	-	Yes
**0087**	+	14.12	+	-	+	+	-	Yes
**0093**	+	19.16	+	-	+	+	-	Yes
**0121**	+	16.79	+	-	+	+	-	Yes
**0130**	+	15.28	+	-	+	+	-	Yes
**0131**	+	20.24	+	-	+	-	-	Yes
**0132**	+	31.04	-	-	-	-	-	Yes
**0140**	+	31.43	-	-	-	-	-	Yes
**0155**	+	33.07	+	-	-	-	-	No
**0156**	-	ND	+	-	-	-	-	No
**0157**	+	33.42	+	-	-	-	-	No

+: positive, -: negative, ND: not detected

Next, we determined the nucleotide sequence of the N gene of the rabies virus to clarify whether alterations of antigenic epitopes recognized by antibodies used in LFDs existed (Table 3). Among the 11 cases positive in the real-time RT-PCR, the nucleotide sequence could not be determined in two cases (ID 0155 and ID 0157), probably due to the low viral copy number. The nucleotide sequences of the remaining 9 cases were completely determined, and the amino acid sequences were identical in 8 cases, except for ID 0121 showing an amino acid substitution from alanine to valine at position 75 (S1 Fig). All discrepant samples in this study were classified as rabies lyssavirus, the same as the one detected in the Philippines in a previous study (S2 Fig).

## Discussion

To achieve “Zero by 30,” a global plan to end human deaths owing to dog-mediated rabies by 2030, the implementation of mass vaccination campaigns for dogs, based on nationwide canine rabies surveillance, is necessary for effective control of dog-mediated rabies in endemic countries [9]. However, animal rabies is inadequately diagnosed in these resource-limited countries because of its low priority for the government or health sector [47]. Therefore, alternative, sustainable, and viable national animal surveillance systems must be considered. We believe that a surveillance system with on-site diagnosis is a suitable option [22,28,33]. An LFD has advantages in on-site diagnosis because it delivers results rapidly and does not require initial costs, such as facilities, equipment, and training. The usefulness of applying LFDs to in-field surveillance in rabies-endemic countries has already been mentioned by Léchenne et al. and Mauti et al. [28,33]. A larger number of studies have demonstrated the high sensitivity and specificity of LFDs [18–28,30–36]. However, the WHO and OIE have not yet recommended using LFD as a confirmatory diagnostic tool, and the LFD results are controversial in some studies [29,37]. Thus, we evaluated three LFDs in parallel with routine dFAT in a rabies-endemic developing country.

In the present study, the sensitivity values of LFDs, excluding the Elabscience kit, were 0.88 (0.80–0.93) and 0.95 (0.88–0.98) for ADTEC and Bionote kits, respectively, and, thus, comparable with that of dFAT. Furthermore, the ADTEC kit DM improved the sensitivity of the ADTEC kit. The high overall sensitivity to detect rabies antigen indicates that both ADTEC and Bionote kits are useful tools for the identification of infected dogs in control programs. The sensitivity of this study was higher than that reported by Klein et al. (0%–62%) [37]. They concluded that LFDs were not suitable for diagnostic applications because the results were variable among facilities. The samples used in our study were submitted on ice or frozen, and most of the samples were shipped within 3 days after death (89.1%). Previous studies evaluating Bionote kits have suggested that fresh samples collected in the field were more sensitive than long-term stored samples [29,37]. Léchenne et al. compared the results of the Bionote kit with those of dFAT in field laboratory settings and indicated that the sensitivity of the LFD approached 100% [33]. In a large-scale survey of LFDs in a government surveillance system, the sensitivity and specificity of the Bionote kit using 209 fresh brains were 0.96 and 0.99, respectively, and the authors concluded that the use of LFDs could help establish field surveillance [22]. It is not clear whether the use of stored samples affected the low sensitivity in the study of Klein et al.; however, LFDs may be more suitable for on-site or immediate use at sample submission than for retrospective application using archival samples.

The components of the LFDs are considered to influence diagnostic accuracy. Here, the Bionote and ADTEC kits showed good detection power, but all results of the Elabscience kit were negative throughout the study. A possible reason for this is what kind of viral antigen is recognized by each LFD. Most rabies detection methods target the viral N protein because it is conserved and abundant in rabies-infected cells and tissues [37,48]. In previous studies, it was demonstrated that Bionote and ADTEC kits recognize the viral N protein [18,34], and in particular, the ADTEC kit recognizes the antigenic sites II and III of the N protein [18]. At the beginning of the study, we included the Elabscience kit as one of the commercially available kits, although its manufacturer did not reveal the target antigens captured by this kit. However, the Elabscience kits turned out to recognize the viral G protein, which can more easily detect free virions and is abundant in saliva. Therefore, the Elabscience kit might show quite low sensitivity by using rabies-infected brain samples of dogs. Although the target antigens and detection limits of ADTEC and Bionote kits were revealed in several reports, relevant information on most of the other available LFDs for rabies diagnosis has not been released. Therefore, clarifying the composition of LFDs is essential for assessing the authenticity of diagnostic kits. Furthermore, there is a need to establish systems that facilitate the distribution of LFDs that have been validated or authorized according to national or international criteria [20,29,37,48].

Five of the 10 false-negative samples in the ADTEC kit tested positive in the ADTEC kit DM. Therefore, direct homogenization with the assay buffer is suitable for the ADTEC kit. In a previous study using the Bionote kit, the method omitting the dilution step with PBS was more sensitive [33]. Originally, the detection efficacy of the ADTEC kit has also been evaluated following direct homogenization in the assay buffer without a prior dilution step in PBS [18]. The advantage of this method is that there is no need to dilute the rabies virus antigen and add reagents except those provided in the kit. Moreover, fewer processing steps will reduce the risk of infection.

ID 0132 and ID 0140 were both negative in the dFAT, but their respective Cq values were above 30, suggesting that the dFAT could not correctly identify the sample as the amount of the viral protein itself was low. However, we might not be able to completely rule out minor contamination of the positive sample during sample collection. Although IDs 0155, 0156, and 0157 were faintly positive in the dFAT performed in the regional laboratory, no fluorescent foci were recognized in repeated observations performed in the RITM that functions as the central laboratory. The Cq values of these three samples were either quite high or under the detection limit, and we could not determine the nucleotide sequences. Based on these reasons, we concluded that IDs 0155, 0156, and 0157 showed false-positive dFAT results performed in RADDL III. Although dFAT is regarded as the gold standard method for rabies diagnosis in humans and animals [33,48], the results are sometimes affected by the examiner’s experience and knowledge [33]. Furthermore, differences in processing methods or in the conjugate also influence the sensitivity and specificity of the test [13,15,49]. To maintain the quality and performance of dFAT in each laboratory, it is absolutely necessary to ensure accurate, reliable, and sustainable results via regular training by a reference laboratory.

There were five discrepancies between dFAT and LFD in the ADTEC kit DM and four in the Bionote kit. Of these, the dFAT results for IDs 0155, 0156, and 0157 were incorrect. However, ID 0055 and ID 0131 showed brightly fluorescent foci in the dFAT and sufficient Cq values in the real-time RT-PCR. As the amino acid sequences of both samples were identical to those of other LFD-positive cases, a mutation in the N gene might not be the cause of the false-negative result. Further studies are required to reveal the cause of this discrepancy. Here, we demonstrated that the LFD sensitivity was as high as 95%, and there were no false-positive results in the Bionote kit and ADTEC kit DM. However, using LFD alone would not be adequate, especially in cases of human exposure; we would like to emphasize that decisions regarding post-exposure prophylaxis cannot be made based only on the result of LFDs. A combination of dFAT or real-time RT-PCR is still required due to the possibility of false-negative results.

One of the factors hampering routine rabies diagnosis in resource-limited countries is the lack of a comprehensive network to transport the samples to diagnostic laboratories [14]. This is the reason why we recommend on-site diagnosis as mentioned above. In the Philippines, where our study was conducted, suspected rabies animals need to be decapitated on-site and then transported to a regional diagnostic laboratory either on ice or frozen. In fact, in Region III, approximately 60% of the suspicious cases were submitted from the province where the regional diagnostic laboratory was located, whereas <50% of suspicious cases were reported from other provinces. Therefore, we deem that on-site diagnosis would be more practical to strengthen diagnostic capacity for resource-limited countries rather than the construction of diagnostic laboratories to improve its accessibility. In addition, on-site diagnosis can reduce the biohazard risks associated with sample transportation, as well as sample degradation that may affect the test result due to long-distance shipments under tropical climate.

Currently, LFDs are regarded as one of the favorable options for on-site rapid diagnosis at a lower initial cost, and animal rabies surveillance in the field using LFDs could be expanded nationwide and be conducted sustainably in resource-limited countries [22]. As demonstrated in the present study and other reports [22,33], some LFDs showed high sensitivity and specificity, which seem to be sufficiently reliable for decision-making of mass dog vaccination and for its post evaluation in endemic countries. Furthermore, by connecting with the Global Positioning System (GPS), the date, time, and location of the diagnosis can be easily registered, and on-site diagnosis can be integrated into data collection using mobile phone applications, which are used in the assessment of mass dog rabies vaccination [10,50]. If the epidemiological situation can be visualized and evidenced through the surveillance system suggested, a government might improve the priority of rabies control measures. As mentioned above, the LFD has advantages with respect to rapid on-site diagnosis, lower initial cost, and high sensitivity and specificity when properly used. Thus, we would like to strongly promote LFDs to be introduced into practical settings in rabies endemic areas in the Philippines. However, before promoting the use of LFDs in a field setting, complete and complementary evaluations, such as multi-site field trials and proficiency tests, and improvements of the LFD quality by the manufacturers, such as modifying protocols to increase the efficiency or batch-to-batch evaluation, are required.

Several limitations and improvements remain to be addressed in this study. First, we did not compare the exact time required for the tests between LFDs and dFAT. From the viewpoint of the time required for on-site testing, LFDs were more effective than dFAT because dFAT in this study was often performed on the next day (47 cases: 25.5%). Second, because the ADTEC kit DM was not performed on-site but tested using the stored sample, the diagnostic performance of the ADTEC kit DM may not have been equally evaluated. Third, most of the samples in this study were obtained from dogs and were conducted at one facility in the Philippines. Therefore, our results may not be applicable to other animal species, including wildlife and other field conditions. Fourth, the costs required to obtain LFD results should be evaluated by comparing them with that of dFAT. Here, the exact cost was not compared because the LFDs for rabies diagnosis are not commercially available and distributed in the Philippines. Furthermore, the usefulness of LFDs should have been evaluated in unequipped areas, such as outdoor laboratories or in the field, not in the regional laboratory, equipped with essential items. From an infection risk perspective, the brain sampling procedure should be improved to be low-risk and straightforward, such as using materials collected without opening the skull [28,48]. Thus, the cost-effectiveness and feasibility of LFDs in outdoor laboratories should be evaluated in future studies to validate whether LFDs are a viable option in diagnosing rabies worldwide.

## Supporting information

S1 Table Summary of dFAT, LFDs (ADTEC, Bionote, Elabscience kits, and ADTEC kit DM), real-time RT-PCR, and virus Cq values in all submitted samples. (XLSX)

S2 Table Characteristics of 156 rabies-suspected animals according to the results of dFAT. (XLSX)

S1 Fig Alignment of N protein amino acid sequences in nine discrepant samples. The N protein amino acid sequence of the PV strain (GenBank GU992322.1) and the nine discrepant samples in this study were aligned and compared by MEGA X. ID 0121 showed an amino acid substitution from alanine to valine at position 75. DDBJ deposit No.LC550027 (ID 0055), LC550026 (ID 0066), LC550025 (ID 0087), LC550024 (ID 0093), LC550022 (ID 0121), LC550021 (ID 0130), LC550020 (ID 0131), LC550019 (ID 0132), and LC550018 (ID 0140).
(TIF)

S2 Fig Phylogenetic tree of the discrepant samples compared with other lyssaviruses constructed using the N gene. The phylogenetic tree was constructed using the N gene (1,353 bp) of rabies lyssavirus in the Philippines, other lyssaviruses, and nine discrepant samples in this study (blue dots). The tree was generated by the neighbor-joint algorithm using the Kimura-2 parameters in MEGA X. The numbers below the branches are bootstrap values for 1,000 replicates.
(TIF)
