# Supplementary figures and images for: Evaluation of the diagnostic accuracy of lateral flow devices as a tool to diagnose rabies in post-mortem animals
Source: PLoS Negl Trop Dis. 2020 Nov 5;14(11):e0008844. doi: 10.1371/journal.pntd.0008844 (PMC7671516; doi:10.1371/journal.pntd.0008844)

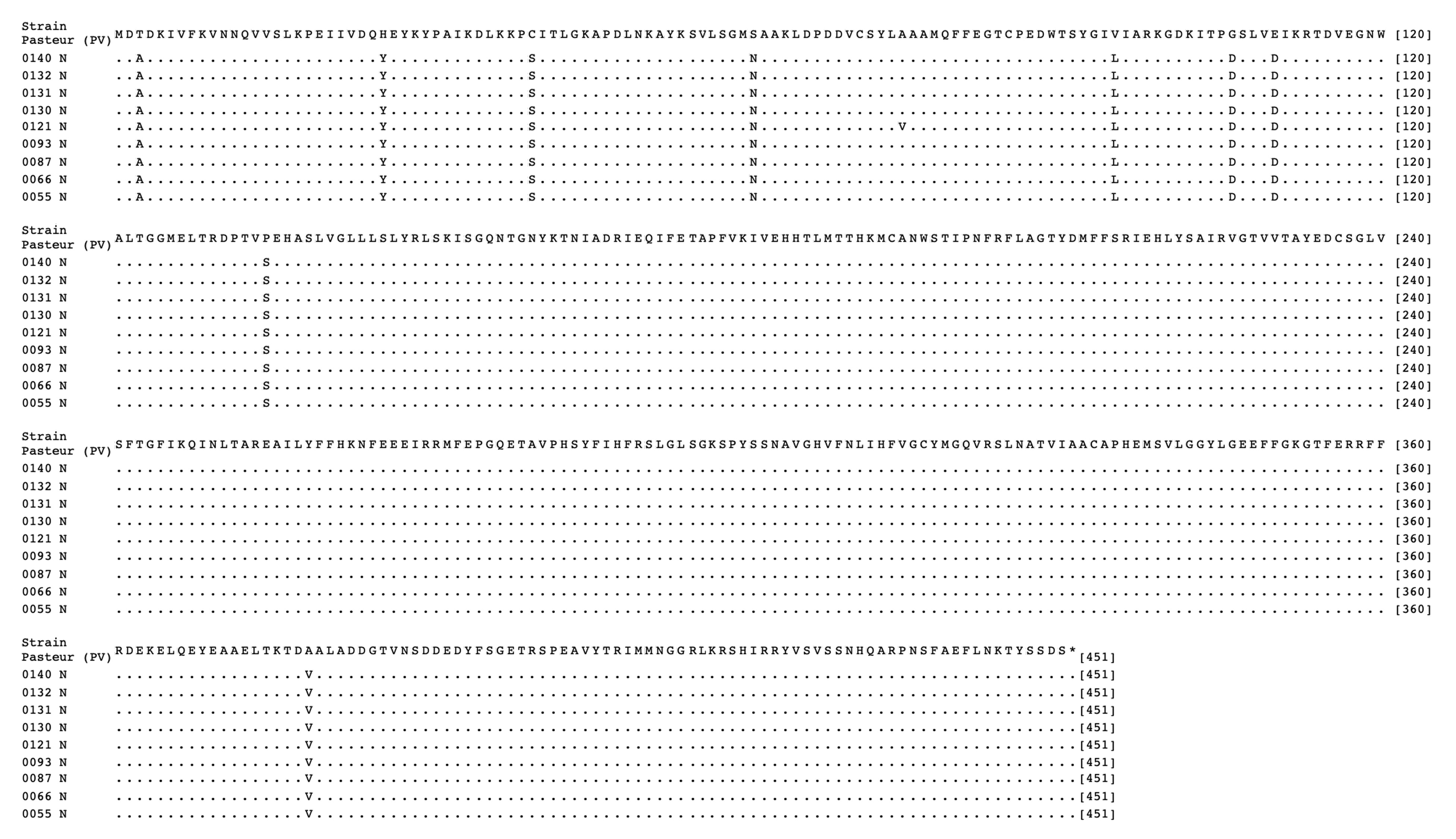

Supplement: S1 Fig — The N protein amino acid sequence of the PV strain (GenBank GU992322.1) and the nine discrepant samples in this study were aligned and compared by MEGA X. ID 0121 showed an amino acid substitution from alanine to valine at position 75. DDBJ deposit No.LC550027 (ID 0055), LC550026 (ID 0066), LC550025 (ID 0087), LC550024 (ID 0093), LC550022 (ID 0121), LC550021 (ID 0130), LC550020 (ID 0131), LC550019 (ID 0132), and LC550018 (ID 0140). (TIF) [file pntd.0008844.s003.tif]

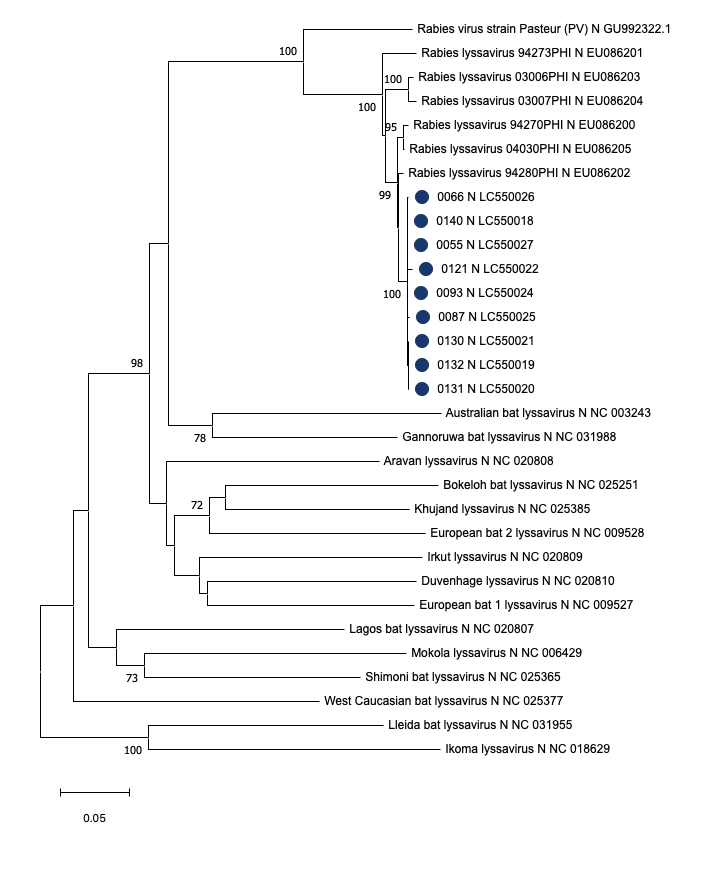

Supplement: S2 Fig — The phylogenetic tree was constructed using the N gene (1,353 bp) of rabies lyssavirus in the Philippines, other lyssaviruses, and nine discrepant samples in this study (blue dots). The tree was generated by the neighbor-joint algorithm using the Kimura-2 parameters in MEGA X. The numbers below the branches are bootstrap values for 1,000 replicates. (TIF) [file pntd.0008844.s004.tif]
